# High‐frequency repetitive transcranial magnetic stimulation ameliorates memory impairment by inhibiting neuroinflammation in the chronic cerebral hypoperfusion mice

**DOI:** 10.1002/brb3.3618

**Published:** 2024-07-15

**Authors:** Huihui Zou, Shilin Bao, Xinrun Chen, Xianju Zhou, Shaotian Zhang

**Affiliations:** ^1^ Department of Neurology, Neuroscience Center Southern Medical University Hospital of Integrated Traditional Chinese and Western Medicine, Southern Medical University No. 13 Shi Liu Gang Road, Haizhu District Guangzhou 510315 China; ^2^ Department of Neurology, Neuroscience Center Sourthern Medical University Hospital of Integrated Traditional Chinese and Western Medicine, Southern Medical University No. 13 Shi Liu Gang Road, Haizhu District Guangzhou 510315 China; ^3^ Department of Neurology General Hospital of Southern Theater Command, Chinese People's Liberation Army Guangzhou China

**Keywords:** bilateral carotid stenosis, chronic cerebral hypoperfusion, cognitive impairment, high‐frequency repetitive transcranial magnetic stimulation, neuroinflammation, neuroplasticity

## Abstract

**Background:**

High‐frequency repetitive transcranial magnetic stimulation (HF‐rTMS) has been found to ameliorate cognitive impairment. However, the effects of HF‐rTMS remain unknown in chronic cerebral hypoperfusion (CCH).

**Aim:**

To investigate the effects of HF‐rTMS on cognitive improvement and its potential mechanisms in CCH mice.

**Materials and methods:**

Daily HF‐rTMS therapy was delivered after bilateral carotid stenosis (BCAS) and continued for 14 days. The mice were randomly assigned to three groups: the sham group, the model group, and the HF‐rTMS group. The Y maze and the new object recognition test were used to assess cognitive function. The expressions of MAP‐2, synapsis, Myelin basic protein(MBP), and brain‐derived growth factors (BDNF) were analyzed by immunofluorescence staining and western blot to evaluate neuronal plasticity and white matter myelin regeneration. Nissl staining and the expression of caspase‐3, Bax, and Bcl‐2 were used to observe neuronal apoptosis. In addition, the activation of microglia and astrocytes were evaluated by fluorescence staining. The inflammation levels of IL‐1β, IL‐6, and Tumor Necrosis Factor(TNF)‐α were detected by qPCR in the hippocampus of mice in each group.

**Results:**

Via behavioral tests, the BCAS mice showed reduced a rate of new object preference and decreased a rate of spontaneous alternations, while HF‐rTMS significantly improved hippocampal learning and memory deficits. In addition, the mice in the model group showed decreased levels of MAP‐2, synapsis, MBP, and BDNF, while HF‐rTMS treatment reversed these effects. As expected, activated microglia and astrocytes increased in the model group, but HF‐rTMS treatment suppressed these changes. HF‐rTMS decreased BCAS‐induced neuronal apoptosis and the expression of pro‐apoptotic protein (Caspase‐3 and Bax) and increased the expression of anti‐apoptotic protein (Bcl‐2). In addition, HF‐rTMS inhibited the expression of inflammatory cytokines (IL‐1β, IL‐6, and TNF‐α).

**Conclusions:**

HF‐rTMS alleviates cognitive impairment in CCH mice by enhancing neuronal plasticity and inhibiting inflammation, thus serving as a potential method for vascular cognitive impairment.

## Graphical abstract

This study highlights the role and mechanisms of high‐frequency repetitive transcranial magnetic stimulation (HF‐rTMS) in chronic cerebral hypoperfusion. HF‐rTMS stimulation has been shown to attenuate cognitive impairment in mice, which is attributed to its enhancement of behavioral functions in mice by inhibiting glial cell activation, alleviating neuronal, damage, regulating synaptic proteins, and alleviating white matter damage.

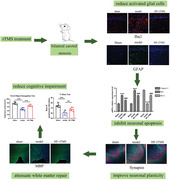

## 1 INTRODUCTION

A sustained decrease in cerebral blood flow CBF) in the cortex, white matter, and the hippocampus leads to chronic cerebral hypoperfusion (CCH), inducing metabolic, cognitive impairment, and neuronal damage (Otori et al., 2003). Neuronal functions are key regulators that are often affected in cognitive impairment (Cubelos & Nieto, 2010). Previous studies have reported that impaired neuronal signaling, conveyed by morphological and density changes in neuronal synapses and dendrites, lead to cognitive impairment after CCH (Chung et al., 2015). In addition, neuroinflammation has been identified as an important mechanism contributing to the pathophysiology of cognitive dysfunction (Belkhelfa et al., 2018). Activation of glial cells and overproduction of inflammatory cytokines after hypoxia lead to reduced blood flow, white matter damage, and cognitive dysfunction (Wada‐Isoe et al., 2004). White matter damage is not merely the result of chronic hypoxia, but also is induced and maintained by pro‐inflammatory environment (Farkas et al., 2007). Currently, there are no known effective treatment options for vascular cognitive impairment (VCI) other than reducing vascular risk factors. Thus, searching for effective treatments to alleviate cognitive impairment has aroused great interest (Liu et al., 2019).

Repetitive transcranial magnetic stimulation (rTMS) is introduced by Barker et al. (1985) as a non‐invasive brain stimulation technique to cause many changes in the brain. Low‐frequency rTMS (LF‐rTMS) (≤1 Hz) produces inhibitory effects, while high‐frequency rTMS (HF‐rTMS) (> 1 Hz) has excitation effects (Klomjai et al., 2015). Currently, rTMS are widely used in clinical treatment for neurological and psychological disorders (Elinos‐Báez et al., 2003; Guse et al., 2010). rTMS have been reported to activate kinase cascades associated with activity‐dependent synaptic plasticity (Fujiki et al., 2020). rTMS, on the other hand, cause extensive long‐term changes in neuronal function, including Long‐term potentiation(LTP)‐ and long‐term depression(LTD)‐like effects as well as intrinsic changes in neurons (Cirillo et al., 2017). More interestingly, TMS is used to map motor and cognitive functions, explore neural networks to regulate brain activity with electrical potential therapeutic purpose (Lanza et al., 2022; Pennisi et al., 2015). However, the underlying mechanisms of improving cognitive impairment after CCH mediated by rTMS have not been elucidated. Therefore, we aimed to explore the effect of HF‐rTMS and its possible mechanisms in the CCH mice. We postulated that rTMS modulates hippocampal synaptic plasticity and relieves neuroinflammation, thereby ameliorating cognitive impairment. Importantly, the findings from this study provide valuable experimental evidence to support the clinical utilization of rTMS as a potential therapeutic approach for patients suffering from VCI.

## 2 METHODS

### 2.1 Animals and model of CCH

Male C57BL/6J mice (8–10 weeks, weight 23–26 g) were provided by the Animal Center of Southern Medical University. They were placed in specific pathogen‐free environments in 12‐h light/12‐h dark cycles, allowing free access to water and food. As previously reported, bilateral carotid stenosis (BCAS) induced CCH (Cirillo et al., 2017). Specifically, ice anesthetized with isoflurane, both common carotid arteries (CCA) were exposed through a midline incision in the neck. Gently lifted one side of CCA and placed it below the carotid bifurcations in the microcoil (0.18‐mm diameter, 2.5 mm length, Sawane Spring) between the rings. The microcoil was wound by rotating around the CCA. The same procedure was performed for the CCA on the left side. The sham mice underwent the same surgical procedure without the implantation of microcoil. The mortality rate was about 8%. All experimental procedures and animal care were conducted in accordance with the Experimental Animal Management Committee of Southern Medical University.

### 2.2 rTMS Treatment

The mice were divided into three groups: (a) the sham group (*n* = 8); (b) the model group with sham stimulation (*n* = 8) (the coil was about 5 cm away from the surface of the brain and therefore unable to produce effective magnetic stimulation) and (c) the model group with HF‐rTMS (*n* = 8). Wild‐type C57BL/ 6J mice were placed in rTMS confinement (C. Zhang et al., 2019). A 70 mm figure‐eight coil (YIRUIDE, 3.0 Tesla) was placed in the center of the animal's head one day after BCAS surgery. The parameters of HF‐rTMS delivery were as follows: 20 Hz frequency, stimulation intensity at 20% 1‐s train duration, inter‐train interval of 14 s, and 20 trains per session. Each session of HF‐rTMS consisted of 1600 pulses/day delivered within 20 min and for 14 consecutive days (once a day in the morning) (S. Zhang et al., 2023).

### 2.3 Y‐maze test

After 14 days of magnetic stimulation, the Y maze test was performed in a Y‐shaped maze consisting of three arms (35 cm length × 10 cm width × 10 cm height) at 120µ angles to each other. During the test, all three arms were opened and the mice were allowed to move freely from the end of the same starting arm for 10 min. Sprayed 75% ethanol, cleaned and air dried the maze for 3 min between each test. Over the course of multiple entries, normal mice showed a tendency to visit the new arm in the maze rather than the recently visited arm. Manually recorded the order of the arm entries. An entry was recorded when all four limbs of the mouse were inside the arm. Spontaneous alternation was defined as entering all three arms in a continuous selection. The percentage of spontaneous alternation was calculated as the ratio of the actual number of possible alternations to the maximum number of possible alternations. If less than eight arms were entered throughout the test period, the animal was excluded from the statistical analysis to avoid the low input times that may affect spontaneous alternate scores.

### 2.4 Object recognition task

The new target recognition test was conducted as before. First, each mouse was put into one square box (29 cm length × 47 cm width × 30 cm height) for 10 min without any object to adapt to the environment. Then, the mice were placed in the test box with two identical objects and allowed to explore for 10 min. One hour later, one of the two identical objects in the test box was replaced by another new object. Once again, the test mice were placed in the test box and allowed to explore freely for 5 min. The sniffing time of the tested mice toward both objects in the test phase was defined as the sniffing behavior when the nose tip was facing toward the object and the distance from the object was within 2 cm. Time for exploring objects was determined by two researchers who were blind to the experiment.

### 2.5 Western blot

On day 14 after magnetic stimulation, all mice were sacrificed, removed the brain and homogenized the hippocampus which was separated from the brain. The same amount of protein (50 µg/ lane) was separated by SDS‐PAGE and then electrophoretically transferred to polyvinylidene fluoride membrane. The membranes were sealed in 5% milk powder for 1 h and incubated overnight at 4µC with primary antibody diluted at 1:1000 against brain‐derived growth factors (BDNF) (Abcam). The membrane was then incubated at room temperature for 1 h with the secondary antibody conjugated with horseradish peroxidase diluted at 1:1000 (Abcam). Protein bands were detected using an ein verbessertes chemolumineszentes(ECL) plus chemiluminescent Regent kit (Bedford Millibo).

### 2.6 Immunofluorescence staining

The mice were euthanized on day 14 after magnetic stimulation and infused with phosphate‐buffered saline (PBS) with saline and 4% formaldehyde. The brains were removed and fixed in 4% formaldehyde at 4µC for 24 h, then dehydrated in 30% sucrose solution. After cryopreservation, coronal sections (4 µm thick) of the frozen brain were prepared, followed by immunofluorescence. After incubation in blocking solution (2% normal goat serum, 3.0% Triton X‐3 in PBS) for 2 h, slices were incubated in a combination of primary antibodies, including MAP‐2 (Abcam), NeuN (Abcam), Synapsis (Abcam), MBP (Abcam), IBA1 (Abcam), and GFAP (Abcam). The slices were then incubated in a mixture of fluorescent secondary antibodies (Alexa 488/Alexa 594 conjugated mouse/Rabbit IgG). All images were acquired using a fluorescence microscope (Carl Zeiss) and analyzed using ImageJ (National Institutes of Health, Bethesda).

### 2.7 Nissl staining

After behavioral tests, the brain was obtained according to the procedure described by immunofluorescence assay. After the sections were dewaxed, they were soaked in triple distilled water for 3 min and stained with methyl violet staining solution for 30 min. The slides were rinsed with distilled water, differentiated with distilled water (Beyotime) solution for 5 s, dehydrated with gradient alcohol solution, transparented with xylene, and then sealed with neutral glue. Finally, the changes in the number of neurons at the same location in the hippocampus were observed using an optical microscope.

### 2.8 Quantitative real‐time PCR

The mouse hippocampus was extracted for RNA extraction on day 14 after magnetic stimulation. The extraction steps were as follows: Trizol (15596026, Ambion) was used to split the tissue, chloroform was added, the nuclear protein was fully decomposed and then centrifuged. Then, the supernatant was thoroughly mixed with isopropyl alcohol. After centrifugation, the supernatant was discarded and 75% ethanol was added to clean the precipitation. Reversed transcribed into cDNA by PrimeScript RT reagent Kit following the manufacturer's guides. Relative mRNA expression levels were performed with SYBR Green detection system. The PCR was performed in a thermal cycler as follows: initial denaturation at 95µC for 10 min, 40 cycles of 95µC for 1 min, and annealing at 60µC for 30 s. The following primers were used:

Caspase‐3 forward: 5′‐CCGCTTATAACTGTTGCT
Caspase‐3 reverse: 5′‐TTCCCAGCGGTCCGCTTCAT‐3′
Bax forward: 5′‐CCCAGAGGCGGGGTTTCA‐3′
Bax reverse: 5′‐GGAAAAAGACCTCTCGGGGG‐3′
Bcl‐2 forward:5′‐CATATCTGTTTCGAGAATCA‐3′
Bcl‐2 reverse:5′‐CACCCGTTTCTCCGATAAGCA‐3′
IL‐1β forward:5′‐GAAATGCCACCTTTTGACAGTG‐3′
IL‐1β reverse:5′‐GAAATGCCACCTTTTGACAGTG‐3′
IL‐6 forward:5′‐TAGTCCTTCCTACCCCAATTTCC‐3′
IL‐6 reverse:5′‐TTGGTCCTTAGCCACTCCTTC −3′
TNFα forward:5′‐CCTGTAGCCCACGTCGTAG‐3′
TNFα reverse: 5′‐GGGAGTAGACAAGGTACAACCC‐3′
GAPDH forward:5′‐ATGACTCTACCCACGGCAAG‐3′
GAPDH reverse:5′‐TACTCAGCACCAGCATCACC‐3′

RNA levels were assayed using the “ΔΔ CT method” for relative expression.

### 2.9 Statistical analysis

Data analysis was performed using SPSS v.11.0 (SPSS Inc.). The data were analyzed by one‐way analysis of variance and the significance of the effect of HF‐rTMS treatment on BCAS mice was determined. When appropriate, post hoc comparisons were made using the least significant difference test or the Dunnett T3 test. Unless otherwise stated, *p*‐values <.05 are considered significant. All data were expressed as SEM ± mean value.

## 3 RESULT

### 3.1 HF‐rTMS rescues BCAS‐induced cognitive impairment mice

**FIGURE 1:**
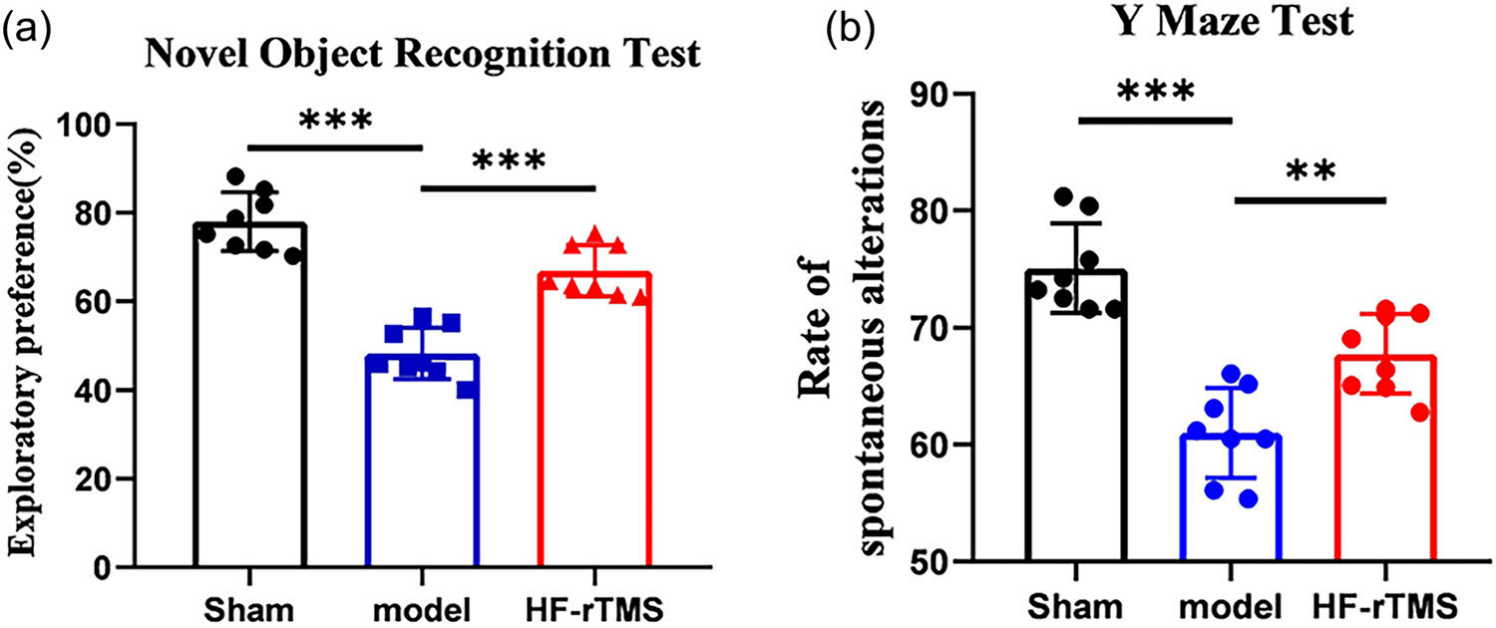
High‐frequency repetitive transcranial magnetic stimulation (HF‐rTMS) ameliorates bilateral carotid stenosis (BCAS)‐induced cognitive impairment. (a) Percentage of time spent on new object of object recognition task. (b) Percentage of correct alterations in the Y‐maze test. The data are presented as the mean ± standard deviation (*n* = 8). **p < .01; ***p < .001.

To investigate the effects of HF‐rTMS on BCAS‐induced cognitive impairment in mice, we examined memory function using novel object recognition task. Compared with the sham group, the model mice showed significant memory impairment (*p* < .001), which was specifically manifested as a significantly reduced preference rate for new objects (*p* < .001) (Figure 1a). However, HF‐rTMS treatment significantly alleviated memory impairment (*p* < .001) (Figure 1a). Similarly, the percentage of correct spontaneous alternations in the model mice was also reduced, while HF‐rTMS intervention significantly reversed these changes (*p* = .0023) (Figure 1b). These results suggested that HF‐rTMS treatment ameliorates memory impairment induced by CCH.

### 3.2 HF‐rTMS inhibits neuronal apoptosis in the hippocampus

**FIGURE 2:**
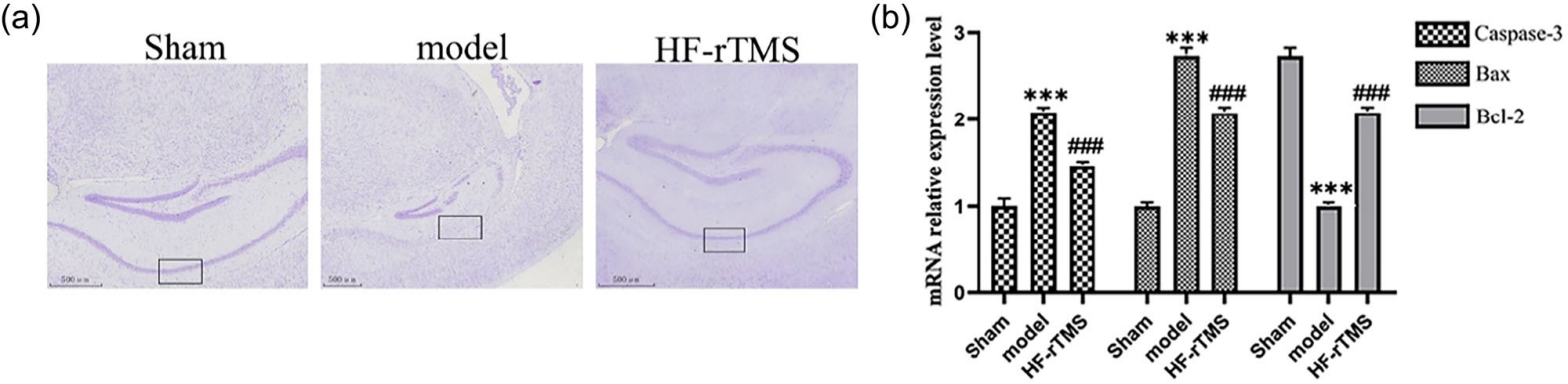
(a) The changes in the morphology of hippocampal neurons in mice were evaluated by Nissl staining (scale bar, 500 µm). (b) Relative expression of Caspase‐3, Bax, and Bcl‐2 mRNA in hippocampal tissues of in three groups of mice. The data are presented as the mean ± standard deviation (*n* = 3). ***p < .001.

Previous studies indicated that rTMS prevents neuronal death and thus improves neuronal survival (Chervyakov et al., 2015), we wanted to the effect of HF‐rTMS on neuronal apoptosis induced by CCH. The morphology of neurons in the sham group was full and the arrangement was regular by Nissl staining (Figure 2a). In the model group, the number of hippocampal neurons decreased significantly, and the arrangement was irregular and vacuolar. However, HF‐rTMS treatment rescued the morphological changes of neurons and increased the number of neurons compared to the model group (Figure 2a).

Next, q‐PCR results showed that in the hippocampus, the expressions of two proapoptotic proteins (Caspase‐3 and Bax) were significantly increased (both *p* < .001), while the expression of antiapoptotic protein Bcl‐2 was decreased (*p <* .001, Figure 2b). Fortunately, HF‐rTMS reversed the pathological changes(all *p* < .001) (Figure 2b). The results suggested that overexpression of proapoptotic protein and the decrease of antiapoptotic protein might affect the cognitive function of mice. Thus, HF‐rTMS may improve cognitive function by regulating these proteins.

### 3.3 HF‐rTMS improves neuronal plasticity after BCAS

**FIGURE 3:**
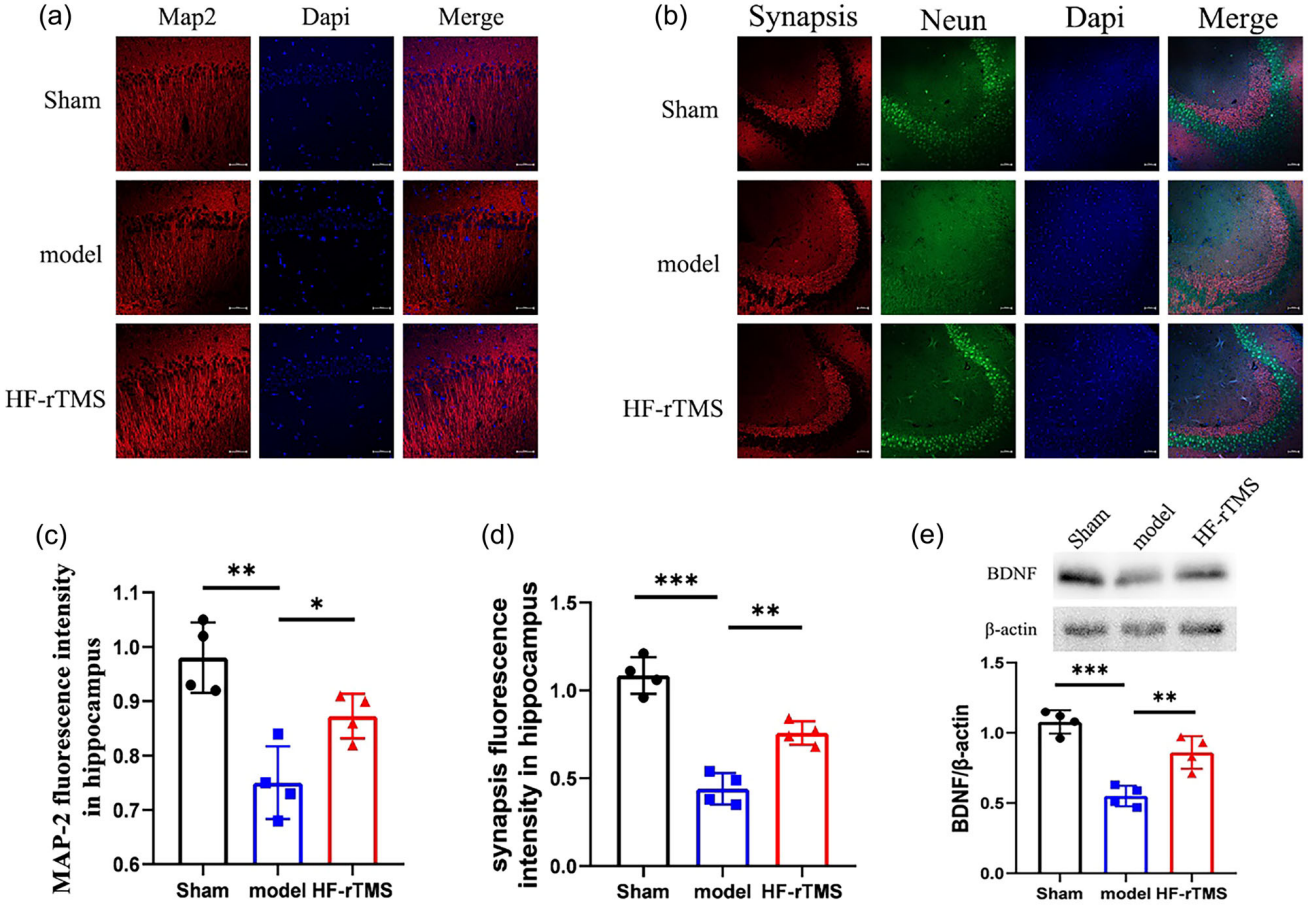
High‐frequency repetitive transcranial magnetic stimulation (HF‐rTMS) improves neuronal plasticity after bilateral carotid stenosis (BCAS). (a) Representative immunofluorescent images showing MAP‐2 (red) in hippocampus. propidium iodide (DAPI) (blue) indicates the nucleus. Scale bar: 20 µm. (b) Representative immunofluorescence images showing colocalization of synapsis (red) and NeuN (green) in the hippocampus. DAPI (blue) indicates the nucleus. Scale bar: 50 µm. (c) Quantitative analysis of MAP‐2 by immunofluorescence. (d) Quantitative analysis of synapsis by immunofluorescence. (e) Western blot detection and quantitative analysis of BDNF. The data are presented as the mean ± standard deviation (*n* = 4). *p < .05; **p < .01; ***p < .001.

To determine whether HF‐rTMS affected mouse neuronal plasticity after BCAS, we used immunofluorescence to label Microtubule‐associated protein 2 (MAP2) or synapsis colocalization with Neun in the hippocampus to assess white matter integrity. MAP2 staining in the model group showed discontinuous expression of MAP2 in the hippocampus and low fluorescence intensity, suggesting the possibility of neuron damage (*p* = .0026, compared to the sham group; Figure 3a,c). In contrast, the hippocampus in the HF‐rTMS group showed more complete expression of MAP2 (*p* = .0205, compared to the model group; Figure 3a,c). Furthermore, synapsis/Neun dual immunostaining showed blurred nuclear boundaries and irregular synaptophysin patterns in the model group (*p* < .001, compared to the sham group; Figure 3b). However, these irregularities were improved more continuously in the HF‐rTMS group compared to the model group (*p* = .0013, Figure 3d).

Considering the important role of BDNF in synpatic plasticity (Femenía et al., 2012), we determined the level of BDNF in the mouse hippocampus. As expected, we found that BDNF levels were reduced in the model group (*p*<.001) as compared with the sham group, while HF‐rTMS elevated the reduced BDNF level (*p* = .004, Figure 3e).

### 3.4 HF‐rTMS attenuates white matter repair after BCAS damage

**FIGURE 4:**
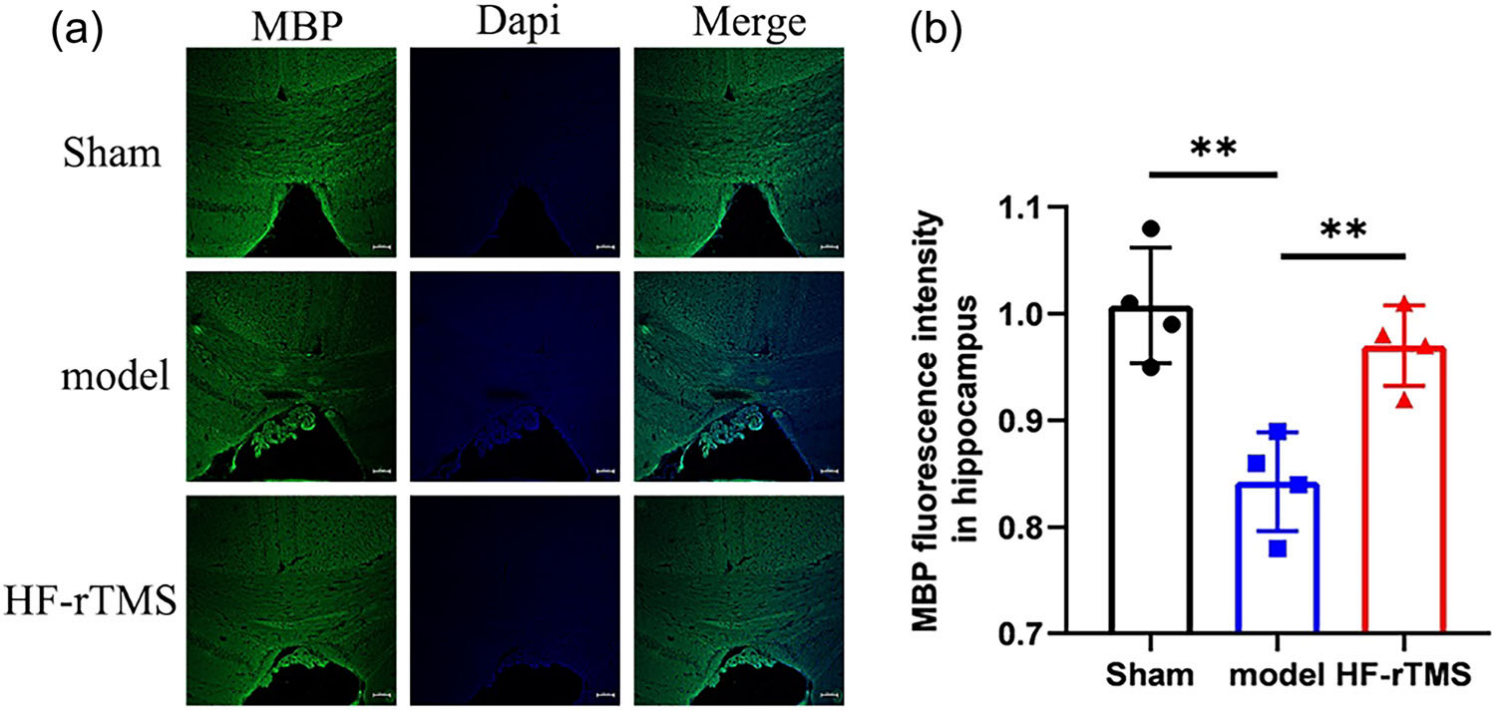
High‐frequency repetitive transcranial magnetic stimulation (HF‐rTMS) improves remyelination of white matter via regulating MBP expression after bilateral carotid stenosis (BCAS). (a) Representative immunofluorescent images showing localization of MBP (green) in callosum. propidium iodide (DAPI) (blue) indicates the nucleus. Scale bar: 100 µm. (b) Quantitative analysis of MBP by immunofluorescence in callosum. The data are presented as the mean ± standard deviation (*n* = 4). **p < .01.

White matter damage is an important manifestation of CCH. Demyelinating axons are an important part of white matter pathology. After white matter injury, the abnormal myelin histopathology in the white matter tract may be manifested as loss of staining (Armstrong et al., 2016). The expression of MBP in corpus callosum was evaluated by immunofluorescence (Figure 4a). Compared with the sham group, MBP staining was weaker in the model group (*p* = .0036, Figure 4b), while HF‐rTMS treatment enhanced MBP expression (*p* = .0052, Figure 4b).

### 3.5 HF‐rTMS reduces activated glial cells after BCAS injury

**FIGURE 5:**
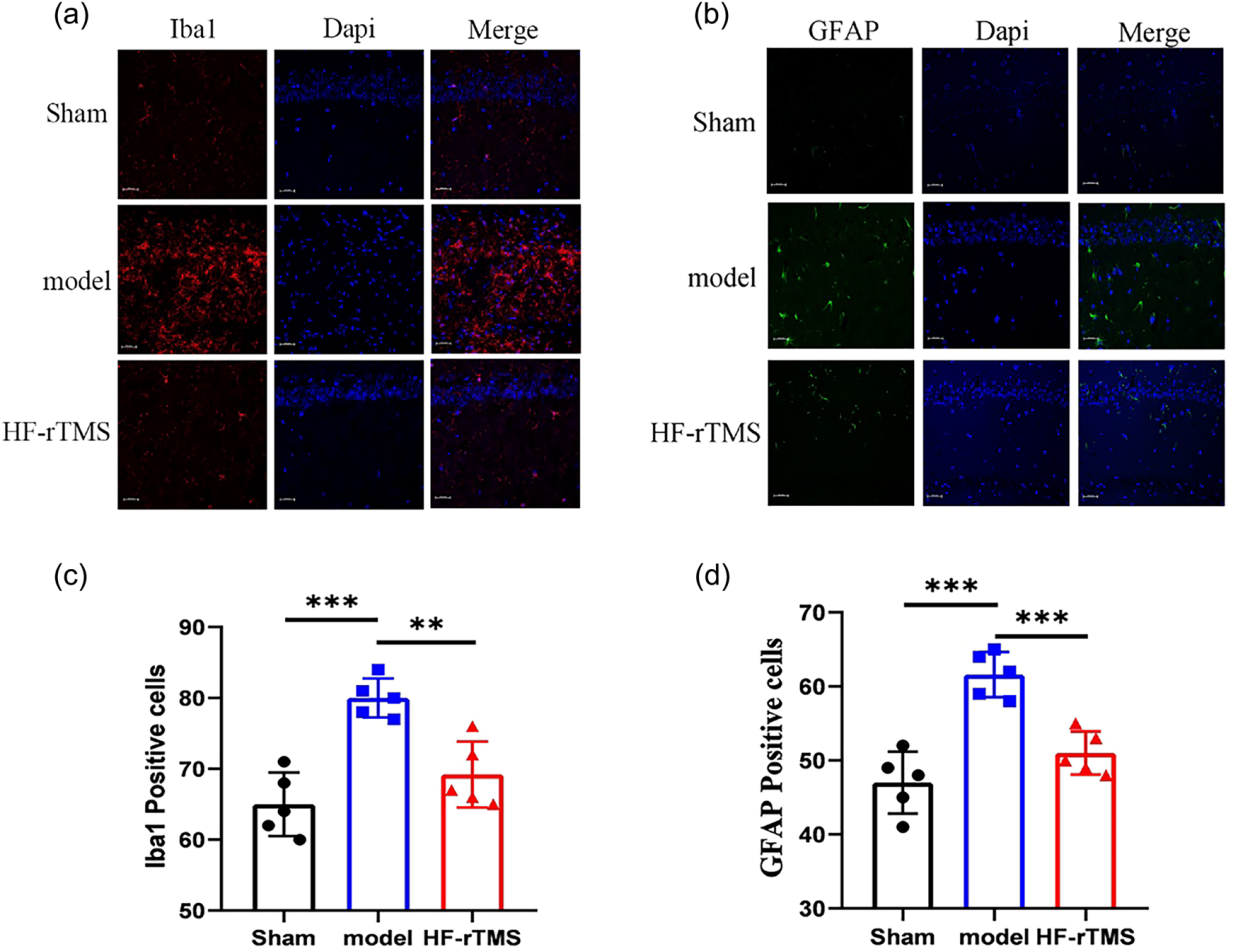
High‐frequency repetitive transcranial magnetic stimulation (HF‐rTMS) attenuates bilateral carotid stenosis (BCAS)‐induced activated glial cells in the mouse hippocampus. Representative photomicrographs of the immunofluorescence analysis of (a) microglia (IBA‐1‐positive cells) and (b) astrocytes (GFAP‐positive cells) in the hippocampal after HF‐rTMS treatment and (c and d) their respective statistical analysis (scale bars: 20 µm; *n* = 4). **p < .01; ***p < .001.

Neuroinflammation is an important factor in cognitive impairment (Alam et al., 2018). The function of microglia is to maintain the homeostasis of the central nervous system. We evaluated the function of microglia cells by immunostaining the molecular marker of microglia, ion calcium‐binding junction molecule 1 (IBA1) (Figure 5a). IBA1 staining showed that there were more positive microglia in the hippocampus of the model group than that of the sham group, with larger cell bodies and more branches (*p* < .001, Figure 5a), while these changes were less significant in the HF‐rTMS group than in the model group (*p* = .0021, Figure 5c).

Astrogliosis is characteristic of BCAS‐induced white matter lesions. We analyzed the changes of astrocytes in the hippocampus after BCAS. A small number of GFAP cell counts were detected in the sham group, compared to the model group, showing higher GFAP intensity and increased GFAP cell counts, significantly increased cell volume (*p* < .001, Figure 5b,d). These changes were alleviated after HF‐rTMS treatment (*p* = .0021). Overall, these data suggested that HF‐rTMS reduces BCAS‐induced microglial activation and astrocyte proliferation, probably preventing BCAS‐induced demyelination.

### 3.6 HF‐rTMS inhibits the level of pro‐inflammatory cytokines after BCAS

**FIGURE 6:**
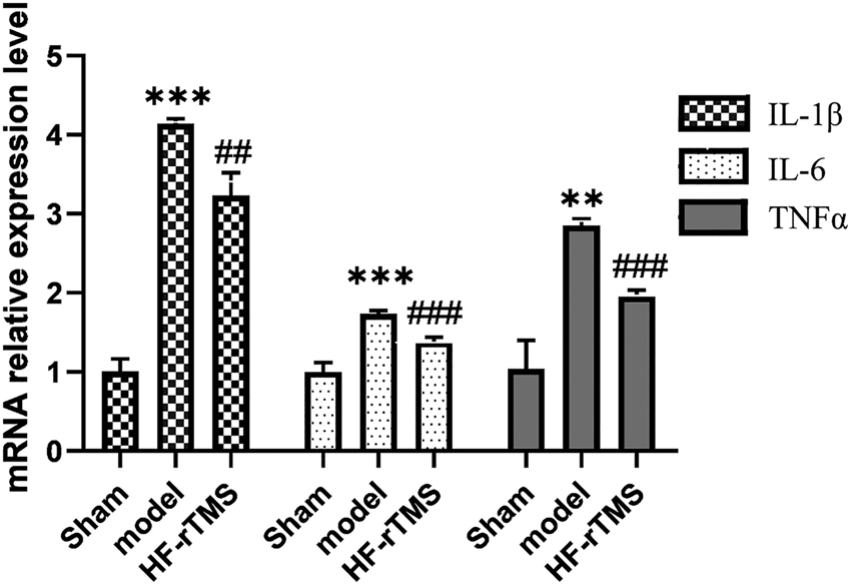
High‐frequency repetitive transcranial magnetic stimulation (HF‐rTMS) reduces the level of proinflammatory cytokines induced by bilateral carotid stenosis (BCAS). (a) Quantification of the data shows mRNA expression levels of IL‐1β, IL‐6, and, TNF‐α (*n* = 3). **p < .01; ***p < .001.

To investigate whether HF‐rTMS reduced the levels of pro‐inflammatory factors in the hippocampus, we measured the expression levels of pro‐inflammatory cytokines such as IL‐1β, IL‐6, and TNF‐α (Figure 6). q‐PCR results showed that the levels of IL‐1β, IL‐6, and TNF‐α were significantly increased in the model group (*p* < .001). The levels of IL‐1β, IL‐6, and TNF‐α were significantly decreased by HF‐rTMS treatment (*p* < .001). The results showed that HF‐rTMS treatment significantly inhibited the increased levels of pro‐inflammatory cytokines in the BCAS mice.

## 4 DISCUSSION

The main findings of our study demonstrate that HF‐rTMS effectively ameliorates cognitive impairment in mice with CCH. Our results suggest that the therapeutic effects of HF‐rTMS may be mediated through multiple mechanisms, including the inhibition of neuronal apoptosis and reduction of neuroinflammation, ultimately promoting synaptic plasticity and cognitive function.

Our findings are consistent with previous studies investigating the potential neuroprotective and restorative effects of HF‐rTMS on cognitive function in various neurological conditions. Specifically, studies in stroke patients and Alzheimer's disease have shown positive results that HF‐rTMS interventions lead to improvements in cognitive impairment compared to controls (Zong et al., 2020) (Yin et al., 2020). Furthermore, the modulation of apoptosis and inflammation by HF‐rTMS is consistent with previous studies highlighting the importance of these processes in neurodegenerative diseases and vascular cognitive impairment (Han et al., 2017; Taylor et al., 2019).

A systematic review by Chou et al. (2020) has shown that three to five consecutive sessions of rTMS improve memory function and executive function in patients with Alzheimer's disease, lasting up to 12 weeks. Also in a randomized, double‐blind, placebo‐controlled study, rTMS effectively enhances cognitive training in Alzheimer's disease (Bagattini et al., 2020). Interestingly as an adjunct therapy, the improvement lasted for 12 weeks after treatment began (Bagattini et al., 2020).

To further elucidate the potential mechanisms underlying the therapeutic effects of HF‐rTMS on cognitive impairment, we explored its impact on key processes implicated in neurodegeneration and inflammation. Specifically, the Bcl‐2 family, known for its crucial role in nerve cell apoptosis, interacts with caspase 3, a key mediator of apoptosis (Vermeulen et al., 2003; Luo et al., 2022). Our study found that rTMS reduced apoptosis after treatment, suggesting that rTMS may alleviate cognitive impairment primarily by inhibiting neuronal apoptosis. Moreover, considering the growing evidence implicating inflammation in neuronal plasticity and myelin regeneration following brain injury, we investigated the involvement of activated microglia cells in cognitive changes post‐cerebral hypoperfusion (Jiang et al., 2021; Li et al., 2020). Previous studies have shown that rTMS can directly reduce neuronal death in neurons, but also found that rTMS can also act on astrocytes to indirectly reduce neuronal death (Hong et al., 2020).

Linking our results to human pathology, particularly vascular cognitive impairment due to chronic subcortical vascular disease (Lanza et al., 2017), underscores the clinical relevance of our findings. Given the similarities between the mouse model of CCH used in our study and human cerebrovascular pathology, our results suggest that HF‐rTMS may have a therapeutic potential for treating cognitive impairment associated with vascular‐related brain disorders.

Our study contributes to the growing body of evidence supporting the use of rTMS as a promising therapeutic approach for cognitive impairment in vascular‐related brain disorders. By targeting apoptosis, inflammation, and synaptic plasticity, HF‐rTMS holds potential for improving cognitive function and quality of life in affected individuals. However, future studies are warranted to validate our findings in human populations and to optimize rTMS protocols for clinical application. However, it is essential to acknowledge the limitations and caveats of our study. First, while our findings provide insights into the mechanisms underlying the therapeutic effects of HF‐rTMS, further research is needed to deeply elucidate these mechanisms, particularly in human subjects. Again, the translation of our results from animal models to clinical settings requires careful consideration of factors such as treatment protocols, patient characteristics, and potential side effects.

## 5 CONCLUSION

In conclusion, our findings underscore the efficacy of HF‐rTMS in alleviating cognitive impairment in CCH mice. These therapeutic effects might be mediated through suppressing neuroinflammation and enhancing synaptic plasticity, thus improving cognition. The results of this study may provide valuable experimental evidence to support rTMS as a potential treatment for patients with VCI.

## AUTHOR CONTRIBUTIONS

**Huihui Zou**: Conceptualization; data curation; formal analysis; visualization; writing—original draft; writing—review and editing; investigation; methodology; software; validation; resources. **Shilin Bao**: Formal analysis; software; data curation. **Xinrun Chen**: Data curation; software. **Xianju Zhou**: Writing—original draft; supervision; methodology; software; funding acquisition. **Shaotian Zhang**: Data curation; formal analysis; visualization.

## CONFLICT OF INTEREST STATEMENT

The authors declare no conflicts of interest.

### PEER REVIEW

The peer review history for this article is available at https://publons.com/publon/10.1002/brb3.3618.

## Data Availability

The data that support the findings of this study are available from the corresponding author upon reasonable request.
